# Deletion of the hemopexin or heme oxygenase-2 gene aggravates brain injury following stroma-free hemoglobin-induced intracerebral hemorrhage

**DOI:** 10.1186/s12974-016-0490-1

**Published:** 2016-02-01

**Authors:** Bo Ma, Jason Patrick Day, Harrison Phillips, Bryan Slootsky, Emanuela Tolosano, Sylvain Doré

**Affiliations:** Department of Anesthesiology, Center for Translational Research in Neurodegenerative Disease, University of Florida College of Medicine, P.O. Box 100159, Gainesville, FL 32610 USA; Departments of Molecular Biotechnology and Health Sciences, University of Torino, Torino, Italy; Departments of Neuroscience, Neurology, Psychiatry, Psychology and Pharmaceutics, University of Florida College of Medicine, Gainesville, FL 32610 USA

**Keywords:** Fluoro-Jade, Hemorrhagic stroke, Iron, Microglia, Perls, Phagocytosis

## Abstract

**Background:**

Following intracerebral hemorrhage (ICH), red blood cells release massive amounts of toxic heme that causes local brain injury. Hemopexin (Hpx) has the highest binding affinity to heme and participates in its transport, while heme oxygenase 2 (HO2) is the rate-limiting enzyme for the degradation of heme. Microglia are the resident macrophages in the brain; however, the significance and role of HO2 and Hpx on microglial clearance of the toxic heme (iron-protoporphyrin IX) after ICH still remain understudied. Accordingly, we postulated that global deletion of constitutive HO2 or Hpx would lead to worsening of ICH outcomes.

**Methods:**

Intracerebral injection of stroma-free hemoglobin (SFHb) was used in our study to induce ICH. Hpx knockout (Hpx^−/−^) or HO2 knockout (HO2^−/−^) mice were injected with 10 μL of SFHb in the striatum. After injection, behavioral/functional tests were performed, along with anatomical analyses. Iron deposition and neuronal degeneration were depicted by Perls’ and Fluoro-Jade B staining, respectively. Immunohistochemistry with anti-ionized calcium-binding adapter protein 1 (Iba1) was used to estimate activated microglial cells around the injured site.

**Results:**

This study shows that deleting Hpx or HO2 aggravated SFHb-induced brain injury. Compared to wild-type littermates, larger lesion volumes were observed in Hpx^−/−^ and HO2^−/−^ mice, which also bear more degenerating neurons in the peri-lesion area 24 h postinjection. Fewer Iba1-positive microglial cells were detected at the peri-lesion area in Hpx^−/−^ and HO2^−/−^ mice, interestingly, which is associated with markedly increased iron-positive microglial cells. Moreover, the Iba1-positive microglial cells increased from 24 to 72 h postinjection and were accompanied with improved neurologic deficits in Hpx^−/−^ and HO2^−/−^ mice. These results suggest that Iba1-positive microglial cells could engulf the extracellular SFHb and provide protective effects after ICH. We then treated cultured primary microglial cells with SFHb at low and high concentrations. The results show that microglial cells actively take up the extracellular SFHb. Of interest, we also found that iron overload in microglia significantly reduces the Iba1 expression level and resultantly inhibits microglial phagocytosis.

**Conclusions:**

This study suggests that microglial cells contribute to hemoglobin-heme clearance after ICH; however, the resultant iron overloads in microglia appear to decrease Iba1 expression and to further inhibit microglial phagocytosis.

## Background

Intracerebral hemorrhage (ICH) causes severe clinical disability and mortality [1]. During ICH, large amounts of erythrocytes are released into the extracellular spaces in the brain. When erythrocytes are lysed, extracellular hemoglobin is rapidly oxidized from ferrous (Fe^2+^) to ferric (Fe^3+^) hemoglobin (methemoglobin) [2, 3], which, in turn, readily releases heme [4, 5]. The free heme is extremely lipophilic and binds to lipids intercalating into cell membranes, which results in cellular oxidative damage [6, 7]. Understanding how the released heme is removed after ICH is important because excess free heme is highly toxic [8–11]. The hemoglobin and heme scavenger proteins haptoglobin (Hp) and hemopexin (Hpx) contribute to hematoma removal after ICH [12], and Hpx has the highest binding affinity to heme (Kd < 1 pM) [12, 13]. Hp and Hpx have been characterized as a sequential defense system with Hp as the primary protector and Hpx as a backup when Hp has been depleted during severe ICH. Interestingly, recent quantitative analysis defined an exponential relationship between Hp availability relative to hemoglobin and related protective activities, illustrating that large Hp quantities are required to prevent hemoglobin toxicity [14], perhaps because oxidatively modified hemoglobin loses its binding affinity to Hp and CD163 [15]. In contrast, the linear relationship between Hpx concentration and protection defined a highly efficient backup scavenger system during conditions of large excess of free hemoglobin [14]. These together suggest that Hpx could be more critical in hematoma removal after ICH than has been known before. One study also supports that another role of Hpx is to act as an antioxidant after blood-heme overload [16]. The heme-Hpx complex is endocytosed by cells expressing the CD91 receptor [17]. It is noteworthy that the CD91 receptor is highly expressed within the brain on vascular cells, microglia, and neurons [18]. The CD163 receptor is known for the uptake of the Hp-hemoglobin complex and is expressed on activated microglia [19]. In the brain, Hp is almost exclusively synthesized by oligodendrocytes [20], and Hpx is expressed on neurons and microglia [21–23]. Moreover, it has been suggested that Hp expression cannot be induced in the brain. For example, intraperitoneal injection of bacterial endotoxin has been shown to cause a robust increase in Hp expression in peripheral organs and blood serum, but not in the brain [15].

The heme oxygenase (HO) system is responsible for cellular heme degradation to biliverdin, iron, and carbon monoxide. Two main isoforms have been reported to date: homologous HO1 and HO2 are microsomal proteins that share more than 45 % residue identity and catalyze the same reaction. However, the HO1 isoform has been extensively studied mainly for its ability to respond to numerous cellular stresses such as oxidants, hemorrhage, or trauma [24–26]. On the contrary, HO2 has been less studied likely due to its apparent constitutive nature. Nevertheless, its particular abundance in the brain emphasizes the relevance of HO2 function [27, 28]. HO2 is constitutively expressed by most brain cells, notably neurons, and endothelial and glial cells [29–31] and accounts for the majority of HO activity in the brain [32]. Under oxidative stress conditions, HO2 can rapidly degrade heme [33, 34]. Further evidence for this concept came from experiments with HO2-deficient animals, demonstrating its involvement in brain cell damage produced by cerebral ischemia and ICH [35, 36]. HO2 is the abundant isoform in the adult rodent brain and has been detected in the forebrain, hippocampus, midbrain, basal ganglia, thalamic regions, cerebellum, and brain stem [37]. Additional effort is required to further clarify the physiological role of the Hpx-HO system in the brain. Also, it remains to be investigated which cell type plays the major role in hemoglobin clearance after ICH.

As the resident macrophages in the brain, microglial cells are purported effectors of the innate response after injuries. Growing evidence suggests a protective role for microglial activation in central nervous system pathologies, including ICH [38–41]. The possible mechanism underlying the beneficial effects of activated/migrating microglia may be phagocytosis. For example, it has been shown that microglial cells were activated and recruited to newly formed β-amyloid plaques within 1 to 2 days in animal models of Alzheimer’s disease [42]. By stimulating the peroxisome proliferator-activated receptor, it has been demonstrated that activated microglia can promote the removal of hematomas after ICH [38]. Even in the resting state, microglial cells can be active and vigilant in the adult brain, and blood-brain barrier disruption provokes immediate activation of microglia [43].

In general, although Hpx and HO2 are important for the clearance of hemoglobin and heme, little is known about the role that the Hpx-HO system plays after ICH and notably in respect of the microglia phagocytosis properties. In this study, we investigated the role of Hpx and HO2 after ICH, using genetically modified mice that have separate deletions for Hpx and HO2, to establish whether Hpx is a critical factor for hematoma removal after ICH and whether HO2 is required for the removal. We also investigated the unique role of microglia in hematoma removal.

## Methods

### Animals

All procedures were approved by the Institutional Animal Care and Use Committee of the University of Florida. Adult male Hpx knockout (Hpx^−/−^) mice (22–28 g) were descendants of those generated by Dr. Tolosano’s lab [44], and HO2 knockout (HO2^−/−^) mice were generated by Drs. Poss and Tonegawa [45]. The mouse genotype was assayed by polymerase chain reaction and was additionally confirmed by standard Western blot analysis. Hpx^−/−^ and HO2^−/−^ mice were backcrossed into the C57BL/6 background, and matched C57BL/6 mice were used as the wild-type (WT) controls. The knockout mice and the size of their litters were normal overall. No cognition or motor dysfunction was observed. When we examined the gross superficial cerebrovascular anatomy, no detectable changes were observed. Mice had access to food and water ad libitum and were housed under controlled conditions (23 ± 2 °C; 12-h light/dark periods).

### Antibodies

The antibodies used for these studies included mouse monoclonal neuronal nuclei (NeuN; specific for neurons) antibody (Millipore, Billerica, MA), rabbit polyclonal ionized calcium-binding adapter protein 1 (Iba1) antibody (specific for microglial-like cells; Wako Bioproducts, Richmond, VA), and glial fibrillary acidic protein (GFAP) antibody (specific for astrocytic-like cells; DAKO, Carpinteria, CA). Secondary antibodies were conjugated with Alexa-488 (Jackson ImmunoResearch, Inc., West Grove, PA) or labeled with avidin-peroxidase-biotin complex (Vector Laboratories, Inc., Burlingame, CA).

### ICH model

The procedure for preparing murine stroma-free hemoglobin (SFHb) has been described previously [46]. In brief, blood was taken by cardiac puncture in mice. After centrifugation (2500 r.p.m.) for 5 min at 4 °C, the supernatant was removed and the cell pellet was washed three times with sterile saline. Cells were then collected, suspended in sterile saline, and lysed by two freeze-thaw cycles. The sample was then centrifuged, the supernatant was removed, and the hemoglobin concentration was determined spectrophotometrically. SFHb was then diluted with sterile saline to 2 mM (expressed as the concentration of the hemoglobin tetramer), which approximates its concentration in whole blood. It was aliquoted and stored at −80 °C until used. For hemoglobin injection, age- and weight-matched male mice were anesthetized with halothane (3 % initial, 1–1.5 % maintenance) in O_2_ and air (80 and 20 %, respectively). To model hemorrhage, we placed mice in a stereotaxic device (Stoelting, Wood Dale, IL) and introduced a 32-gauge stainless-steel needle through a burr hole into the right striatum at the following stereotactic coordinates: 0.5 mm anterior and 2.0 mm lateral of the bregma, 3.5 mm in depth. We then injected them unilaterally with 10 μL of 2 mM SFHb over a period of 30 min with a microinfusion apparatus. The injection needle was slowly withdrawn 15 min later, and the wound was sutured. Mice in the sham group received sterile saline injection only. Rectal temperature was maintained at 37.0 ± 0.5 °C throughout the experimental and recovery periods. At 24 and 72 h after SFHb injection, behavioral tests were performed and brains were harvested for stroke injury analysis.

### Locomotor activity

Locomotor activities were assessed before ICH and 24, 48, and 72 h after ICH by an automated system (MED Associates, Inc., St. Albans, VT). The mice were placed in four transparent acrylic cages at the same time every day and monitored for locomotor (horizontal activity), rearing (vertical activity), and stereotypy behaviors during a 30-min test period. The results are expressed as an activity ratio of the baseline for each mouse [47].

### Neurological scoring

Neurological deficits were assessed at 24 and 72 h after SFHb injection. An experimenter blind to the mouse genotype scored all mice for neurological deficits with a 24-point neurological scoring system [48]. The tests included body symmetry, gait, climbing, circling behavior, front-limb symmetry, and compulsory circling and whisker response. Each test was graded from 0 to 4, establishing a maximum deficit score of 24. Immediately after the testing, the mice were sacrificed for injury analysis.

### Histology and immunohistochemistry

At 24 and 72 h after ICH, mice were euthanized and perfused transcardially with phosphate-buffered saline (PBS; pH 7.4) and then ice-cold 4 % paraformaldehyde (PFA) in PBS. The brains were removed, postfixed, and cut into 30-μm coronal sections with a cryostat. The mounted sections were stained with cresyl violet to estimate the lesion volume. Six to eight coronal sections, including the entire injured hemorrhagic area, were summed, and the lesion volumes in cubic millimeters were calculated by multiplying the thickness with the measured areas [47]. All slides were scanned using ScanScope CS (Aperio Technologies, Inc., Vista, CA) and analyzed using ImageScope software (Aperio Technologies, Inc.). For immunohistochemistry, free-floating sections or primary microglial cells were rinsed in PBS after fixation and permeabilization and then incubated at room temperature in 5 % donkey or goat serum to block nonspecific binding. All primary antibodies were diluted in PBS and applied overnight at 4 °C. Antibody concentrations were as follows: rabbit anti-Iba1: 1:1000; rabbit anti-GFAP: 1:2000; mouse anti-NeuN: 1:500. Avidin-peroxidase-labeled biotin-complex secondary antibodies (1:1000) and Vectastain ABC and 3,3′-diaminobenzidine (DAB) SK-4100 kits (Vector Laboratories, Inc.) were then used according to the manufacturer’s instructions. When followed with fluorescence staining, the sections were incubated with a secondary antibody conjugated with Alexa-488 (1:1000). After being rinsed, all sections were mounted in DAPI Hardest Reagent (Vector Laboratories, Inc.) under a glass coverslip. To use as negative controls, additional sections were incubated without the primary antibodies. Stained sections were examined with a Nikon TE2000-E Eclipse fluorescence microscope (Nikon Instruments, Inc., Melville, NY); the images were captured and analyzed by SPOT advanced image software (Diagnostic Instruments, Inc., Sterling Heights, MI). To quantify the numbers of positive cells, photos were taken of four regions of interest in each section containing the infarct sites.

### Perls’ iron and Fluoro-Jade B staining

Iron deposition was detected with Perls’ staining for mainly nonheme ferric iron (Fe^3+^) followed by DAB development. Briefly, brain sections or primary microglial cells were washed in PBS after fixation in 4 % PFA and then incubated with Perls’ solution (10 % potassium ferrocyanide and 20 % HCl, equal parts in PBS) for 30 min. After washing in PBS, the sections or microglial cells were incubated with DAB and hydrogen peroxide for 5 min. Brain sections were finally counterstained with hematoxylin for 2 min. The DAB intensification and hematoxylin counterstaining were omitted when brain sections were co-stained with specific cellular markers.

To determine neuronal cell degeneration in brain tissue, Fluoro-Jade B staining was used according to published protocol [49]. In brief, the slides were first immersed in a solution containing 1 % sodium hydroxide in 80 % alcohol for 5 min. This was followed by 2 min in 70 % alcohol and 2 min in distilled water. The slides were then transferred to a solution of 0.06 % potassium permanganate for 10 min and then rinsed in distilled water for 2 min. The staining solution was prepared from a 0.01 % stock solution of Fluoro-Jade B (Histo-Chem, Inc., Jefferson, AR). After 20 min in the staining solution, the slides were washed in distilled water. The dry slides were then cleared by xylene before coverslipping.

Perls’ iron and Fluoro-Jade B-positive cells were counted in three to four fields immediately adjacent to the hematoma in each section. At least three sections per animal over a magnification field of ×400 were averaged and expressed as cells per field. The images of stained sections were captured and analyzed by SPOT image software. Tissue sections were all processed and analyzed by an observer who was blind to the mouse genotype.

### Primary microglial cell cultures

Primary microglial cultures were prepared as described previously [50]. In brief, the mixed cell culture was prepared from postnatal 2- to 4-day-old mice and then maintained at 37 °C and 5 % CO_2_ for 10 to 15 days in Dulbecco’s modified Eagle’s medium (DMEM) containing 10 % heat-inactivated fetal bovine serum (FBS), 50 U/mL penicillin, and 50 μg/mL streptomycin. Microglial cells were collected as floating cells by gentle shaking and used for the following experiments.

### In vitro phagocytosis assay

Primary microglial cells were plated in divided dishes (35 mm, four compartments; Greiner Bio-One, Monroe, NC) for 48 h at a density of 60,000 cells per well in DMEM with 10 % FBS. Latex beads (6 μm, internally dyed with the fluorophore flash green; Polysciences, Inc., Warrington, PA) were preopsonized in 50 % FBS and PBS. Cells were pretreated with SFHb (1 mM) or vehicle for 2 h. Microglial cell media were then replaced with DMEM alone, and preopsonized beads were added to the cells at a concentration of 10 beads per cell. Microglial cells and beads were incubated at 37 °C for 1 h and then subsequently washed with ice-cold PBS. After fixation with 4 % PFA, the images were taken using the EVOS digital inverted fluorescence microscope (Life Technologies, Grand Island, NY).

### Statistics

Data are expressed as mean ± SEM. Prism 5 software (GraphPad) was used for statistical analysis. In all comparisons, a *P* value less than 0.05 was considered significant. The statistical comparisons among multiple groups were made by one-way ANOVA followed by Newman-Keuls multiple comparison tests or two-way ANOVA followed by Bonferroni multiple comparison tests, except for neurologic deficit scores, which were calculated by the nonparametric Kruskal-Wallis test followed by Dunn’s multiple comparison tests. Differences between two groups were determined by unpaired two-tailed Student’s *t* test.

## Results

### Mortality

Overall, injection of 10 μL of SFHb did not cause any mortality. Of note, we had one mouse die following anesthesia before performing the injection.

### Deletion of Hpx and HO2 aggravates brain injury after SFHb injection

**Fig. 1:**
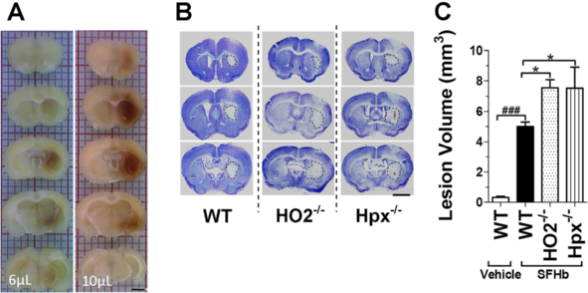
Deleting Hpx and HO2 aggravates brain injury after SFHb injection. Photographs on the *left* are representative brain coronal sections without staining from WT mice illustrating the location of released hemoglobin 5 h after injection with 6 or 10 μL of SFHb (**a**). A separate cohort of age- and weight-matched WT, HO2^−/−^, and Hpx^−/−^ male mice were injected in the striatum with 10 μL of SFHb; brains were then sectioned and stained with cresyl violet 24 h after injection. The lesion perimeter is showed with a *dotted line* (**b**). The quantitative analysis shows that HO2^−/−^ and Hpx^−/−^ mice have markedly larger lesion volume than the WT mice (**c**). *Scale bar*, 2 mm. Values represent means ± SEM. *P < 0.05, ^###^ *P* < 0.001 compared to WT mice, one-way ANOVA followed by Newman-Keuls multiple comparison tests (*n* = 5–6)

Our preliminary studies showed that backflow did not occur when mice were injected over a period of 30 min with 10 μL of SFHb. This concentration of 2 mM SFHb approximates that in whole blood [46]. To define the location and distribution of SFHb, a cohort of mice was injected with 6 or 10 μL of SFHb in the right striatum and sacrificed 5 h after injection. Brain sections revealed that the injected 10 μL of SFHb diffused to the whole striatum, which led to behavioral disability. This condition was reproducible and optimal to assess the removal capacity of the Hpx-HO2 system (Fig. 1a). Thus, we chose the 10-μL injections of SFHb for the following experiments. Cresyl violet staining was used to quantify the lesion volume after injection (Fig. 1b). The results illustrated that 10 μL of SFHb led to a lesion in the striatum at 24 h post-ICH. The Hpx^−/−^ and HO2^−/−^ mice had larger lesion volume compared to their WT controls (Hpx^−/−^, 7.5 ± 1.4; HO2^−/−^, 7.6 ± 0.5 vs. WT, 5.0 ± 0.3 mm^3^; both *P* < 0.05; Fig. 1c). In addition, no obvious lesion was observed in the saline-injected group (data not shown).

### Deletion of Hpx and HO2 aggravates behavioral deficits after SFHb injection

**Fig. 2:**
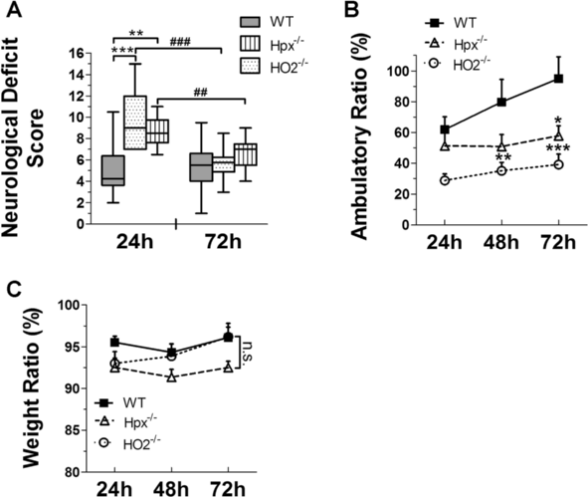
Deleting Hpx and HO2 aggravates behavioral deficits after SFHb injection. Following SFHb injection, an investigator blind to the genotype assessed neurological function with neurological deficit score (**a**) and locomotor activity tests (**b**). All the mice showed recovery from the SFHb-induced injury 24 to 72 h after injection. The neurological deficit scores of HO2^−/−^ and Hpx^−/−^ mice are markedly higher than WTcontrols at 24 h, which suggests that neurological deficits were more severe in HO2^−/−^ and Hpx^−/−^ mice (**a**). In the locomotor test, the ambulatory distance of HO2^−/−^ and Hpx^−/−^ mice was significantly decreased compared with WT controls 72 h after injection (**b**). The body weight of all mice was decreased due to surgery; however, there was no significant difference among different genotypes after injection (**c**). Values represent means ± SEM, *n* = 10–12 per group. *P < 0.05, **P < 0.01, ***P < 0.001 compared to WT mice; ^##^ *P* < 0.01, ^###^ *P* <0.001 compared to 72 h, one-way or two-way ANOVA followed by Newman-Keuls or Bonferroni comparison tests except for neurological deficit scores, which were calculated by the nonparametric Kruskal-Wallis test followed by Dunn’s multiple comparison test

At the same time, behavioral tests were performed blindly on the mice until 72 h after SFHb injection. The data indicated that significant behavioral deficits were caused by injecting 10 μL of SFHb in mice. Moreover, deleting Hpx or HO2 exerted detrimental effects on the neurological deficit score and undermined the locomotor activities in contrast with their WT controls (Fig. 2b). Further, improved performance on behavioral tests over time suggested that the brain injury caused by injected SFHb was recovering from 24 to 72 h postinjection (Fig. 2a, b). In addition, the 10-μL SFHb injection reduced mouse body weight after surgery; however, this was not significant among genotypes and times (Fig. 2c).

### Deletion of Hpx and HO2 causes more iron deposition and neuronal degeneration

**Fig. 3:**
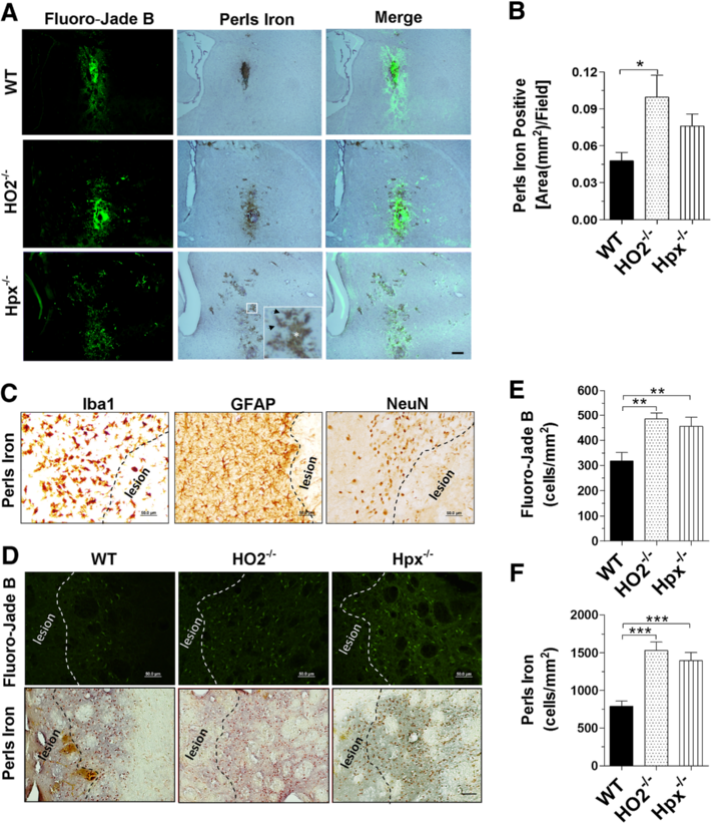
Iron deposition causes neuronal degeneration. Age- and weight-matched WT, HO2^−/−^, and Hpx^−/−^ male mice were injected with 10 μL of SFHb, and brains were sectioned and stained with Fluoro-Jade B and Perls’ iron staining 24 h after injection, respectively. Under microscopy, the positive signals of Fluoro-Jade B and Perls’ iron staining were colocalized partially on continuous neighbor sections. The *inset image* shows that glia-like cells (*arrow head*) were activated around the lesion area (*star*). *Scale bar*, 200 μm (**a**). Quantitative data demonstrated that HO2^−/−^ mice have a larger Perls’ iron-positive area compared to WT controls after injection (**b**). Representative images were shown of the Perls’ iron staining (*blue*) with various cellular markers (*brown*): Iba1 for microglial cells, GFAP for astrocytes, and NeuN for neurons, which illustrated that the Perls’ iron-positive signals were mainly in microglial cells. *Scale bar*, 50 μm (**c**). The degenerating neurons and Perls’ iron-positive cells are shown in coronal sections. *Scale bar*, 50 μm (**d**). The 10-μL SFHb injection produced significantly more degenerating neurons (**e**) and Perls’ iron-positive microglial cells (**f**) in HO2^−/−^ and Hpx^−/−^ mice than those in WT controls. Values represent means ± SEM. *P < 0.05, **P < 0.01, and ***P < 0.001, compared to WT mice, one-way ANOVA followed by Newman-Keuls multiple comparison tests (*n* = 5–6)

It has been reported that the accumulated ferric iron after ICH produced oxidative stress and loss of neurons [9]. To show whether the toxic iron deposition resulted in neuronal degeneration at 24 h postinjection, we performed Perls’ iron and Fluoro-Jade B staining on a series of sections. Interestingly, we found that the two positively stained signals were colocalized on successive sections (Fig. 3a), supporting that iron overload contributes to neuronal degeneration after ICH. Quantitative results demonstrated that HO2^−/−^ mice have a larger Perls’ iron-positive area compared to WT controls after SFHb injection (Fig. 3b). Under light microscopy, we observed two types of Perls’ iron staining: one was diffused and located in the lesion area, which is generally colocalized with the dense positive signals of Fluoro-Jade B, and the other was located inside glia-like cells (Fig. 3a). To better identify the cell type with iron accumulation, we performed the Perls’ iron staining with various cellular markers: Iba1 for microglial cells, GFAP for astrocytes, and NeuN for neurons. The results showed that the Perls’ iron-positive signals were mainly in microglial cells (Fig. 3c), suggesting that microglia would appear to be the main cells for heme clearance after ICH. However, the extracellular diffused irons are toxic and cause neuronal degeneration after ICH. It has been reported that macrophages recycle iron in the liver, spleen, and bone marrow [51]. A recent publication reported that proliferation of local resident microglia rather than recruitment of circulating myeloid cells would be the main source of microgliosis after stroke [52]. Our data are overall consistent with such observation, although we could not entirely exclude the role of infiltrating macrophages.

To further address the effects of Hpx or HO2 deletion on neuronal degeneration and iron overload, we then quantified the Fluoro-Jade B-positive neuronal cells and the Perls’ iron-positive microglial cells around the lesion 24 h postinjection (Fig. 3d). Compared to the WT controls, it was shown that Hpx^−/−^ and HO2^−/−^ mice had more degenerating neurons (Hpx^−/−^, 455 ± 36; HO2^−/−^, 486 ± 23 vs. WT, 318 ± 34 cells/mm^2^; both *P* < 0.01; Fig. 3e) and Perls’ iron-positive cells (Hpx^−/−^, 1394 ± 109; HO2^−/−^, 1532 ± 110 vs. WT, 788 ± 69 cells/mm^2^; both *P* < 0.001; Fig. 3f). Therefore, we concluded that the resident microglial cells in the brain are actively involved in removing iron products after ICH. However, deleting Hpx and HO2 could attenuate this ability because of resultant iron overload in microglia, as indicated by Perls’ staining.

### Deletion of Hpx and HO2 reduces activated microglia after SFHb injection

**Fig. 4:**
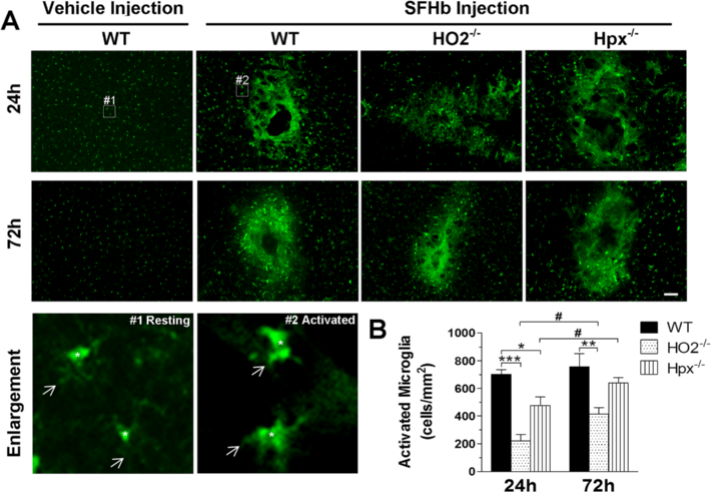
Deleting Hpx and HO2 reduces activated microglia. The distribution and morphology of microglia (Iba1 positive) can be seen in coronal sections collected at 24 and 72 h from WT and Hpx^−/−^ mice (**a**). Activated microglial cells were observed in and around the injury region after the SFHb injection (*#2*, with large cell bodies bearing short and thick processes) in contrast with resting microglia in the vehicle group (*#1*, with small cell bodies bearing ramified long and fine processes). *Stars* indicate cell bodies and *arrows* present cell processes. Quantitative analysis demonstrated that HO2^−/−^ and Hpx^−/−^ mice had less activated microglia at 24 and 72 h than WT mice. In addition, 72 h after injection, their activated microglia cells were significantly increased compared with those at 24 h (**b**). *Scale bar*, 50 μm; values represent means ± SEM.*P < 0.05, **P < 0.01, and ***P < 0.001, compared to WT mice; #P < 0.05 compared to 72 h, one-way or two-way ANOVA followed by Newman-Keuls or Bonferroni comparison tests (*n* = 5–6)

It is known that microglia can be activated to exert migration and phagocytosis. We and others have postulated that activated microglial cells could play the critical role in hemoglobin clearance after ICH [38, 47]. However, activated microglia also release proinflammatory factors and reactive oxygen species, which can cause neuronal toxicity. Therefore, it is intriguing to check the net effect of microglial activation in this context of SFHb injection. Iba1 is widely accepted as a marker to show the resting and activated microglial cells in the brain, with an increased expression level during activation. To assess the effects of Hpx or HO2 knockout on microglial activation, we immunostained brain sections with Iba1 antibody to observe microglial activation/morphology around the lesion 24 and 72 h after SFHb injection (Fig. 4a). The injected SFHb was able to induce microglial activation, and the quantitative data showed that Hpx^−/−^ and HO2^−/−^ mice had much less Iba1-positive cells around the lesion compared to WT controls at 24 h (Hpx^−/−^, 476 ± 62, *P* < 0.05; HO2^−/−^, 220 ± 48, *P* < 0.001 vs. WT, 704 ± 34 cells/mm^2^) and 72 h postinjection (Hpx^−/−^; 640 ± 41, *P* > 0.05; HO2^−/−^; 416 ± 46, *P* < 0.01 vs. WT; 758 ± 95 cells/mm^2^; Fig. 4b), suggesting that iron deposition within microglial cells could potentially reduce the Iba1 expression level after SFHb injection. Additionally, the numbers of activated microglial cells were increasing from 24 to 72 h postinjection, especially in Hpx^−/−^ mice (from 476 ± 62 to 640 ± 41 cells/mm^2^; *P* < 0.05) and HO2^−/−^ mice (from 220 ± 48 to 416 ± 46 cells/mm^2^; *P* < 0.05; Fig. 4b), which was accompanied with the improvement of behavioral tests during the same time period. Therefore, these results support that microglia could play a net protective role in this SFHb-injection ICH model.

### Hemoglobin treatment induces iron deposition and reduces Iba1 expression

**Fig. 5:**
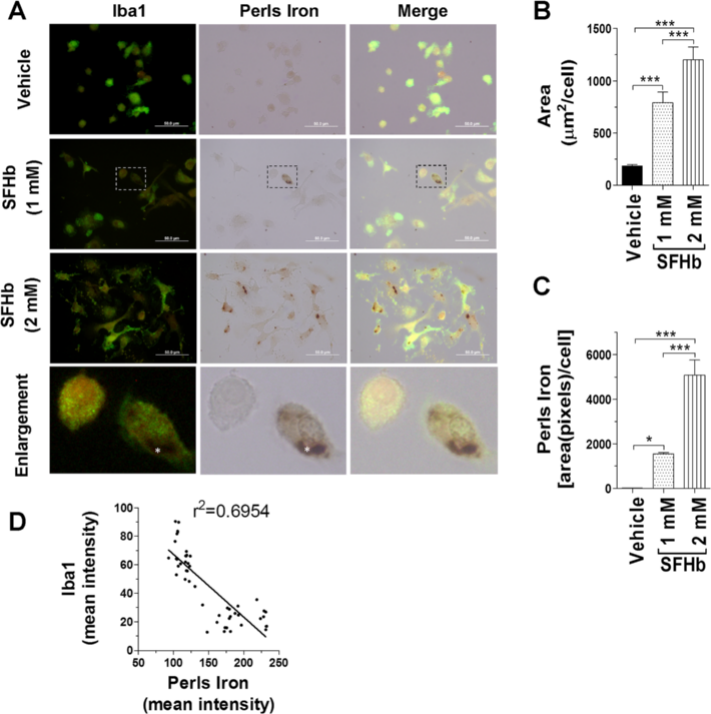
Hemoglobin treatment induces iron deposition and reduces Iba1 expression. The primary microglia derived from WT mice were treated with SFHb for 2 h as indicated concentration. Distribution of Iba1 (*green*) and iron localization (*black*) was shown in microglia by double staining. The cells highlighted within the *dashed box* were demonstrated at a higher magnification in the enlargement. The asterisk (*) indicates that Iba1 signals were absent at the Perls’ iron-positive area (**a**). The *bar graph* shows the significant changes in the size of microglia that are Perls’ iron-positive. SFHb treatment greatly increases microglial size, which is dose-dependent (**b**). The SFHb-treated microglial cells have a larger area of positive Perls’ iron staining, suggesting SFHb-induced iron deposition inside microglia (**c**). The *linear-regression graph* illustrates that Perls’ iron-positive signals are negatively related to Iba1-positive signals, *n* ≥ 50 cells (**d**). *Scale bar*, 50 μm. Values represent means ± SEM. Significant differences between the groups are expressed as follows: *P < 0.05; ***P < 0.001, one-way ANOVA followed by Newman-Keuls multiple comparison tests. The experiment was repeated three times and in *n* ≥ 150 cells

To further address whether microglial cells take up heme-hemoglobin and contribute to hemoglobin clearance, we treated cultured primarily microglial cells with two doses of SFHb for 2 h and then performed the Perls’ iron and Iba1 double staining (Fig. 5a). First, we confirmed that hemoglobin can induce microglial activation. The areas of microglial cells treated with SFHb were markedly bigger than vehicle-treated cells (Fig. 5b). Second, it was observed that SFHb treatment significantly increased the amount of iron accumulated in microglia and that higher SFHb treatment dosage resulted in more iron deposition within microglial cells (Fig. 5c). Further, we found that the accumulated iron in microglia reduced the Iba1 expression level, illustrating a significant negative correlation by linear-regression analysis (Fig. 5d). This observation supports our in vivo data that the injected SFHb may either directly or indirectly decrease the Iba1 expression level and concurrently change Iba1-positive microglia to Perls’ iron-positive microglia.

### Hemoglobin treatment inhibits microglial phagocytosis

**Fig. 6:**
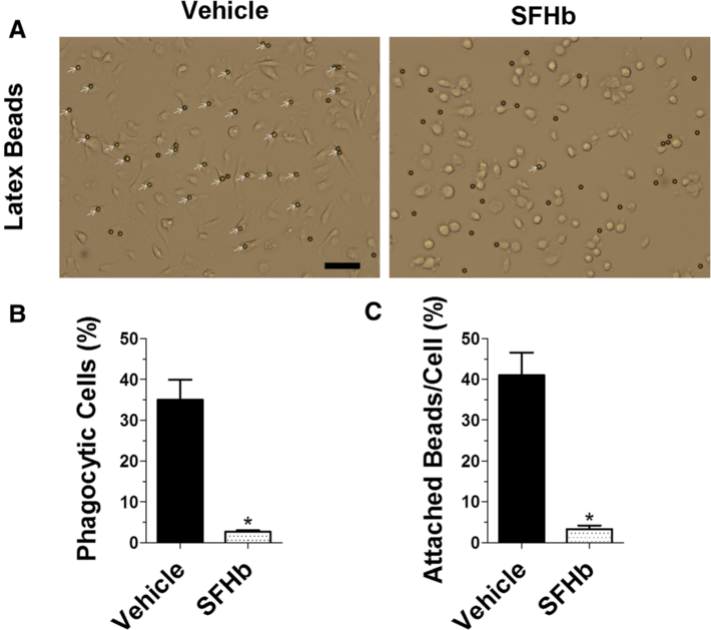
Hemoglobin treatment inhibits microglial phagocytosis. Primary microglial cells were treated with SFHb (1 mM) or vehicle for 2 h and then incubated with fluorescent latex beads (diameter 6 μm) for 1 h. Representative images were taken and *white arrows* illustrate the attached beads to microglial cells (**a**). *Bar graphs* show that the hemoglobin pretreatment markedly reduces the percentage of phagocytic microglial cells (**b**) and the numbers of attached beads (**c**). *Scale bar*, 50 μm. Values represent means ± SEM. Differences between two groups were determined by unpaired two-tailed Student’s *t* test. The experiment was repeated three times and in *n* ≥ 150 cells

Iba1 is known to play an important role in microglia migration and phagocytosis [53, 54]. To determine the effect of iron overload on microglial phagocytosis, we treated primary microglial cells with SFHb (1 mM) or vehicle for 2 h and then incubated them with fluorescent latex beads for 1 h (Fig. 6a). The results showed that the hemoglobin pretreatment markedly reduced the percentage of phagocytic microglial cells (Fig. 6b) and the numbers of beads attached by microglia (Fig. 6c). This may suggest that following ICH, hemoglobin degradation within cells would affect microglial phagocytosis and delay resultant hematoma removal.

## Discussion

By using the 10-μL SFHb-injection model, we found that Hpx or HO2 deletion leads to aggravated brain injury and Perls’ iron deposition, which was consistent with the behavioral results showing the neurological and locomotor disability of the Hpx^−/−^ and HO2^−/−^ mice. Furthermore, by performing double staining of Perls’ iron with various cellular markers, we showed that the Perls’ iron-positive cells were mainly Iba1-positive microglial cells. Using mouse primary microglial cultures, we also observed that the SFHb dose-dependently increased Perls’ iron deposition within these cells and, interestingly, iron accumulation appeared to negatively correlate with Iba1 staining. Finally, using similar cultures, we observed that the rate of beads being phagocytosed within SFHb-treated microglia was drastically attenuated. These data suggest that (a) microglial cells contribute to hemoglobin-heme clearance after ICH; (b) however, the resultant heme/iron overloads in microglia appears to decrease Iba1 expression and further inhibit microglial phagocytosis. Therefore, microglial cells may have a sort of threshold for their hemoglobin-heme degradative capacity. Above this level, their activity, such as phagocytosis, is severely compromised.

The disparate effects of HO2 knockout have been shown in whole-blood and collagenase-injection models, which indicate the complexity in rodent ICH models and the necessity of a simplified ICH model [55, 56]. Here, in the 10-μL SFHb-injection model, the hemoglobin diffuses throughout the mouse striatum and produces a gradient distribution to induce the activation of microglia, which could be optimal to evaluate the hemoglobin-removal capacity of the Hpx-HO system. Also, hemoglobin injection excludes the intrinsic inflammatory influence of the erythrocyte debris and recombinant collagenase in whole-blood and collagenase-injection models. In our preliminary test, we had confirmed that minimal backflow happened over a very slow 30-min period of injection before withdrawing the needle. In addition, Hpx-deficient mice are viable and fertile [44]. Nevertheless, Hpx^−/−^ mice were shown having iron deposits in oligodendrocytes [57, 58]. The Hp protein and mRNA levels are comparable in serum between naïve Hpx^−/−^ and WT mice; however, after hemolytic stimulus, Hpx^−/−^ mice showed persisted Hp levels in the circulation, suggesting compensatory expression of Hp induced by hemolysis [44]. It is well known that iron reacts with lipid hydroperoxides to produce free radicals. Free radicals attack DNA, lipids, and proteins, causing oxidative brain injury. In this study, the degradation of the injected SFHb caused two types of iron deposition in brain tissues: one appearing to be intensely diffused iron deposition in the lesion area and the other suggested to be iron overload in microglial cells. The results demonstrated that the distribution of Perls’ iron staining was consistent with that of neuronal degeneration shown by Fluoro-Jade B staining, which suggested that iron overload mainly contributes to neuronal degeneration after SFHb injection. Under microscopy, we found that the Perls’ iron-positive cells that were glia-like migrated around the lesion area. To further determine which cell type mainly contributes to this hemoglobin clearance and cellular iron accumulation around lesions, we performed double staining of various cellular markers with Perls’ iron and showed that microglia mainly contribute to the clearance of hemoglobin after ICH. This result was supported by the study from Keep’s group [59], and it was also reported by Koeppen’s group that most iron-positive cells around intracerebral hematoma were microglia [60].

It could be concluded that whether activated microglia exert protective or toxic effects might be context dependent, determined by both injury severity and duration [61, 62]. For example, the exogenous application of microglia was shown to protect against different types of ischemic injury in vivo [63–65] and in vitro [40]. On the other hand, brain microglial activation and its related inflammatory response had been shown to confer neurotoxicity in various models of neurodegeneration [66–69]. In this SFHb-injection model, compared to the toxicity of the large amount of hemoglobin, the harmful effects of the proinflammatory cytokines released from the activated microglial cells might remain minor within the experimental time frame and the beneficial potential of the neuroprotective factors might be limited. Therefore, we suggest that under our experimental protocol, it was the effect of microglial phagocytosis that contributed to hemoglobin/heme clearance and behavioral improvement over time.

An interesting observation here was the suggestion that the iron overload appeared to reduce the Iba1 expression level, which could lead to reduced microglial phagocytosis. Also, it may be the reason why more Perls’ iron-positive staining microglia were accompanied with less Iba1-positive microglia around the lesion area in HO2^−/−^ and Hpx^−/−^ mice. As mentioned, Iba1 proteins have been described as playing an essential role in microglia migration and phagocytosis [53, 70]. Thus, we speculate that the effect of HO2 deletion may be increasing the vulnerability of microglia to hemoglobin, potentially similar to the effect already reported for HO1 deficiency [71]. Hpx^−/−^ mice could change the way heme was delivered to microglia and neurons from a controlled, receptor-based mechanism to uncontrolled intercalation into membranes and subsequent oxidative injury. Thus, it was not entirely surprising that it would result in a deleterious effect on microglia and neurons, while increasing iron staining.

## Conclusions

Our findings suggest that microglial cells contribute to hemoglobin-heme clearance after ICH; however, the resultant iron overloads in microglia appear to decrease Iba1 expression and further inhibit microglial phagocytosis that warrants further investigation.

## Abbreviations

DAB: 3,3′-diaminobenzidine
DMEM: Dulbecco’s modified Eagle’s medium
FBS: fetal bovine serum
GFAP: glial fibrillary acidic protein
HO: heme oxygenase
HO2: heme oxygenase 2
HO2^−/−^: HO2 knockout
Hpx: hemopexin
Hpx^−/−^: Hpx knockout
Iba1: ionized calcium-binding adapter protein 1
ICH: intracerebral hemorrhage
PBS: phosphate-buffered saline
PFA: paraformaldehyde
SFHb: stroma-free hemoglobin
WT: wild type
